# A systematic review of the safety information contained within the Summaries of Product Characteristics of medications licensed in the United Kingdom for Attention Deficit Hyperactivity Disorder. how does the safety prescribing advice compare with national guidance?

**DOI:** 10.1186/1753-2000-6-2

**Published:** 2012-01-10

**Authors:** Nicola Savill, Chris J Bushe

**Affiliations:** 1 Medical Department, Eli Lilly and Company Ltd, Lilly House, Priestley Road, Basingstoke, Hants, RG24 9NL, UK

**Keywords:** ADHD, guidelines, summary of product characteristics, drug safety, NICE, SIGN

## Abstract

**Background:**

The safety of paediatric medications is paramount and contraindications provide clear pragmatic advice. Further advice may be accessed through Summaries of Product Characteristics (SPCs) and relevant national guidelines. The SPC can be considered the ultimate independent guideline and is regularly updated. In 2008, the authors undertook a systematic review of the SPC contraindications of medications licensed in the United Kingdom (UK) for the treatment of Attention Deficit Hyperactivity Disorder (ADHD). At that time, there were fewer contraindications reported in the SPC for atomoxetine than methylphenidate and the specific contraindications varied considerably amongst methylphenidate formulations. In 2009, the European Medicines Agency (EMA) mandated harmonisation of methylphenidate SPCs. Between September and November 2011, there were three changes to the atomoxetine SPC that resulted in revised prescribing information. In addition, Clinical Guidance has also been produced by the National Institute for Health and Clinical Excellence (NICE) (2008), the Scottish Intercollegiate Guidelines Network (SIGN) (2009) and the British National Formulary for Children (BNFC).

**Methods:**

An updated systematic review of the Contraindications sections of the SPCs of all medications currently licensed for treatment of ADHD in the UK was undertaken and independent statements regarding contraindications and relevant warnings and precautions were then compared with UK national guidance with the aim of assessing any disparity and potential areas of confusion for prescribers.

**Results:**

As of November 2011, there were seven medications available in the UK for the treatment of ADHD. There are 15 contraindications for most formulations of methylphenidate, 14 for dexamfetamine and 5 for atomoxetine. Significant differences exist between the SPCs and national guidance part due to the ongoing reactive process of amending the former as new information becomes known. In addition, recommendations are made outside UK SPC licensed indications and a significant contraindication for methylphenidate (suicidal behaviours) is missing from both the NICE and SIGN guidelines. Particular disparity exists relating to monitoring for suicidal and psychiatric side effects. The BNFC has not yet been updated in line with the European Union (EU) Directive on methylphenidate; it does not include any contraindications for atomoxetine but describes contraindications for methylphenidate that are no longer in the SPC.

**Conclusion:**

Clinicians seeking prescribing advice from critical independent sources of data, such as SPCs and national guidelines, may be confused by the disparity that exists. There are major differences between guidelines and SPCs and neither should be referred to in isolation. The SPC represents the most relevant source of safety data to aid prescribing of medications for ADHD as they present the most current safety data in line with increased exposure. National guidelines may need more regular updates.

## Background

Attention Deficit Hyperactivity Disorder is a commonly diagnosed disorder affecting around 3-9% of school aged children and young people in the UK; the worldwide pooled prevalence rate of ADHD in the same population is estimated at 5.29% [1,2] The number of prescriptions written for ADHD medications in England alone has risen significantly over time with an almost doubling of rates from 220,000 prescriptions in 1998 to 418,300 prescriptions in 2004 [3]. Data from the UK General Practice Research Database demonstrated that the prevalence of prescriptions in a sample of 1,636 patients increased 6.23-fold between 1999 and 2006 [4]. Associated with increased diagnosis and treatment of ADHD is a need for improved understanding and awareness of the safety considerations detailed within the contraindications and warnings for the various drugs licensed in the UK for treatment of ADHD.

At the time of writing, there were eight medications licensed in the UK to treat ADHD. These medications were granted a marketing authorisation (license) by the UK regulatory agency, the Medicines and Healthcare products Regulatory Agency (MHRA) (formerly the Medicines Control Agency) following review of required efficacy and safety data. The MHRA is a government agency established in 2003 and is responsible for ensuring that medicines and medical devices are effective and are acceptably safe. Safety information is monitored from a variety of sources and, if required, risk benefit assessments are conducted. The MHRA initiated a risk: benefit assessment for atomoxetine in 2006.

A draft SPC is submitted within the application for a product license and is then finalised in conjunction with the Marketing Authorisation Holder (MAH) and the regulatory agency with the purpose of enabling safe and appropriate prescribing. The totality of the data reviewed by the regulatory agency and which subsequently informs the SPC far exceeds that which is published in the peer-reviewed scientific literature. In summary, the SPC is the agreed statement of known facts about a given pharmaceutical compound at a particular point in time and it is critical that it is reviewed and amended regularly as new information emerges.

Guidelines on the preparation and maintenance of SPCs are laid out by the European Commission (EC) and contraindications are defined as 'situations where the medicinal product must not be given for safety reasons' [5]. Statements in the Contraindications section of the SPC are categorical and thus hierarchically stronger than those in either the Special Warnings and Precautions for Use or the Undesirable Effects sections.

The conduct of pharmacovigilance for medicines for paediatric use requires special attention and reporting by multiple stakeholders of potential adverse events is of increasing importance [6]. One such stakeholder is the MAH who is required to submit Periodic Update Safety Reports (PSURs) to the national competent authorities for all marketed products. The purpose of the PSUR is to report an overview of worldwide safety experience, summarise safety data within a given time period, and provide a critical evaluation of the risk: benefit balance of the product. The PSUR captures all reports of drug safety events considered 'related' to the product whether they are reported spontaneously during clinical use or in clinical trials or appear in the published literature. The PSUR is submitted on a periodic basis, every 6 months following approval for a period of 2 years after initial placement on the market, then on a yearly basis for the next 2 years (and at the renewal) and subsequently every 3 years. However, the competent authority may require a more frequent submission in specific cases. The review of PSURs for atomoxetine led to three recent changes in the prescribing information all within approximately 3 months. The first change updated the Undesirable Effects to include anaphylactic reactions, depression and depressed mood, anxiety and tics and pheochromocytoma was added as a contraindication in line with the SPCs for methylphenidate. The next variation resulted in amended wording to the Hepatic Warnings and Undesirable Effects to reflect severe liver injury cases arising from increased exposure as a result of post-marketing surveillance reports. The third variation arose from clinical trial analysis of heart rate and blood pressure data which has culminated in strengthened wording relating to cardiovascular effects and the requirement to conduct baseline and ongoing monitoring of pulse and blood pressure and contraindications for patients with severe cardiovascular and cerebrovascular disorders as is the case for methylphenidate. This last change was accompanied by a Dear Healthcare Professional Letter issued to healthcare professionals in the UK in December 2011.

Because monitoring can and does lead to important safety changes the SPC can be regarded as the current and updated 'ultimate guideline' for safe and effective use of that compound. Other worldwide regulatory agencies have similar views. The US Food and Drug Administration (FDA) which is responsible for protecting the public health by assuring the safety, efficacy and security of drugs in the United States considers the communication of risks and benefits through its product labelling as "the cornerstone of risk management efforts for prescription drugs" [7]. The SPC is thus crucial in achieving safe prescribing.

Although the entire SPC is important, the sections that relate to Contraindications (Section 4.3) and Special Warnings and Precautions for Use (Section 4.4) remain the most critical with respect to safety. Healthcare professionals, however, are not always fully aware of the content of SPCs, how it has been derived or how to access them.

In April 2008, the authors presented a systematic review of the Contraindications sections of the eight medicines authorised in the UK at that time for the treatment of ADHD in children [8]. Among the findings was that atomoxetine, a non-stimulant treatment for ADHD, had far fewer contraindications than stimulant medications (most commonly, formulations of methylphenidate). This review also identified highly varying contraindications among the different methylphenidate formulations despite the similar nature of the methylphenidate ingredient. Whilst the reasons for this are unclear, it may result from different MA holders submitting separate PSURs for their respective methylphenidate containing medications. The number of contraindications for the different presentations of methylphenidate ranged from 10-20 per formulation. All forms of methylphenidate were contraindicated in marked anxiety/tension, diagnosis/family history of Tourette's syndrome, severe angina, arrhythmias and hyperthyroidism. Glaucoma was the only contraindication common to all medications, stimulant and non-stimulant and atomoxetine was the only treatment with no psychiatric, cardiac or neurological contraindications at that time.

The European Medicines Agency (EMA) is responsible for the scientific evaluation of medicines developed by pharmaceutical companies for use in the European Union and constantly monitors the safety of medicines through a pharmacovigilance network, and takes appropriate actions if adverse drug reaction reports suggest that the risk: benefit balance of a medicine has changed since it was authorised. In 2009, the results of a full risk: benefit assessment of methylphenidate-containing medications by the EMA's Committee for Medicinal Products for Human Use (CHMP) under Article 31 of Directive 2001/83/EC (amended) was published. This review was conducted due to concerns over cardiovascular risks (hypertension, heart rate increases and arrhythmias) and cerebrovascular risks (migraine, cerebrovascular accident, stroke, cerebral infarction, cerebral vasculitis and cerebral ischaemia). Following the review, the Committee concluded there was no need for an urgent restriction to the use of methylphenidate-containing medicines, but that new recommendations on prescribing and on pre-treatment screening and ongoing monitoring of patients were needed to maximise the safe use of these medicines. Because information about their safety was not consistent across the EU, the CHMP concluded that the product information of all methylphenidate-containing medicines authorised in the Member States should contain the same information [9]. As a result, it was recommended that approximately 15 identical contraindications to methylphenidate be included in the SPC of all methylphenidate formulations and harmonised across the EU [10].

In general, prescribers in the UK are expected to follow national guidelines to assist prescribing and for individual cases may refer to the SPC. The aim of this systematic review is to compare the contraindications for all medications for ADHD in the UK and to compare prescribing advice and safety information given in the most commonly referred to UK ADHD guidelines with the Contraindications and Special Warnings and Precautions for Use sections of the SPCs to identify any disparity that may potentially complicate prescribing decisions.

## Methods

All SPCs for medications licensed in the UK for the treatment of ADHD were accessed on the electronic Medicines Compendium (eMC) website (November 2011) and the contraindications in each were tabulated. Any SPCs not available on the eMC website were obtained directly from the MAH. Contraindications were defined individually and copied as specified from each SPC. Where contraindications were markedly overlapping, they were categorised together by the authors. The process of tabulating contraindications was carried out by the lead author and checked and reviewed by the second author.

The safety advice and recommendations provided in two UK National Guidelines, SIGN and NICE, were reviewed by the authors. NICE clinical guidelines make recommendations about the treatment and care of people with specific diseases and conditions within the National Health Service in England and Wales and represent the view of the Institute. They are based on publically available data. SIGN Guidelines aim to provide a framework for evidence based assessment and management of ADHD/Hyperkinetic Disorder (HKD) which can be applied with a local multidisciplinary and multiagency approach" in Scotland [11].

Both guidelines were also compared with the safety information contained within the SPCs. Any disparity between the National Guidelines and the SPCs relating to indications, contraindications and warnings/precautions was tabulated.

## Results

### Medications

As of October 2011, there were seven medications licensed and available in the UK for the treatment of ADHD: atomoxetine (Strattera^®^), dexamfetamine, two short-acting preparations of methylphenidate (Ritalin^®^, Medikinet^®^) and three long-acting preparations of methylphenidate (Concerta XL^®^, Equasym XL^® ^and Medikinet XL^®^). Concerta^® ^XL has one SPC listed on the eMC website for the 18-36 mg strength and one for the 27 mg strength, although the contraindications for all strengths are the same. Medikinet^® ^has one SPC listed on the eMC website for the short-acting preparation and two SPCs for the long-acting preparations, one covering the 10-40 mg strengths (Medikinet XL^®^) and one for the 5 mg strength (Medikinet XL^® ^5 mg). The two Medikinet XL^® ^SPCs are identical with respect to contraindications. Thus, Table 1 (Licensing Information) lists the nine separate SPCs but Table 2 (Contraindications) lists seven SPCs, with the two Concerta XL^® ^SPCs and the two Medikinet XL^® ^SPCs each being treated as one. One further medication, Equasym, was removed from the eMC website in September 2011 and is no longer available or marketed in the UK. Equasym was not considered in this review.

**Table 1:** Licensing Information for Available Medications

Drug	Date of Authorisation (Renewal Date)	**Date of Last Substantive**† Revision to SPC Text	Reason for Revision
Strattera^®^	May 2004 (May 2009)	27 September 2011	Addition of contraindication and changes to wording on cardiovascular effects, allergic events, depression, tics, anxiety and overdose (Sections 4.3, 4.4, 4.8, 4.9 and 5.1)
		3 November 2011	Change to wording on hepatic function (Sections 4.4 and 4.8) Addition of comparator data trial outcomes (Section 5.1) Addition of cytochrome P450 wording to Pharmacokinetic properties (Section 5.2)
		25 November 2011	Addition of cardiovascular contraindication and change to wording on cardiovascular status (Sections 4.2, 4.3 and 4.4)

Ritalin^®^	October 1997 (April 2004)	12 May 2011	Expanded wording in the aggression and hostility warnings/overdose section relating to effects of titration from long acting preparations (Sections 4.4, 4.9 and 10)

Concerta^® ^18-36 mg	February 2002 (June 2007)	17 June 2011	Change to prescribing information relating to continuation use in adulthood (Sections 4.2, 4.4, 4.8, 5.1 and 10)

Concerta^® ^27 mg	March 2007	17 June 2011	

Equasym XL^®^	February 2005	10 October 2011	Bruxism added as a common adverse drug reaction under Undesirable Effects (Section 4.8)

Medikinet^®^	February 2007 (November 2008)	3 February 2010	Numerous sections updated as per article 31 Referral

Medikinet XL^®^ 10-40 mg	February 2007 (November 2008)	3 February 2010	Update as per article 31 Referral

Medikinet XL^® ^5 mg	January 2011	24 August 2011	Addition to eMC of new SPC for new product

Dexamfetamine**	October 1992	Not available	Not applicable

Source: http://www.medicines.org.uk/emc/ Accessed 14 November 2011

eMC: electronic Medicines Compendium; SPC: Summary of Product Characteristics

*Relicensed to Shire

**March 2010: Sold to Auden McKenzie who do not subscribe to the eMC

†Substantive defined as any changes to prescribing information which affect safe or effective use of the medication

**Table 2:** Contraindications Listed on the SPC for Medications Licensed in the UK for ADHD

Category	Non-stimulant	Stimulants					
**Brand Name**	**Strattera**^**®**^	**Ritalin**^**®**^	**Concerta**^**®**^ **(18-36/27 mg)**	**Equasym XL**^**®**^	**Medikinet**^**®**^	**Medikinet XL**^**®**^ **(5/10-40 mg)**	**Not applicable**

**Generic Name**	**Atomoxetine**	**MPH**	**MPH**	**MPH**	**MPH**	**MPH**	**Dexamfetamine**

**General**							

Hypersensitivity	X	X	X	X	X	X	X

(Narrow angle) Glaucoma	(X)	X	X	X	X	X	X

With MAIOs/Within 14 days	X	X	X	X	X	X	X

Pregnancy/lactation							X

Pronounced anacidity of the stomach						X	

With H2-receptor blockers/antacid therapy						X	

Sucrose/lactose intolerances and related disorders							X

Porphyria							X

Pheochromocytoma	X	X	X	X	X	X	

**Psychiatric**							

Hyperexcitability							X

Severe depression		X	X	X	X	X	

Anorexia nervosa		X	X	X	X	X	

Psychopatho-logical personality structure		X	X	X	X	X	

Suicidal tendency		X	X	X	X	X	

Psychotic symptoms		X	X	X	X	X	

Schizophrenia		X	X	X	X	X	

Mania/bipolar disorder		X	X	X	X	X	

Severe mood disorders		X	X	X	X	X	

Drug or alcohol abuse							X

**Endocrinological**							

Hyperthyroidism/thyrotoxicosis		X	X	X	X	X	X

**Cardiac**							

Patients with severe cardiovascular **§**or cerebrovascular **†**disorders whose condition would be expected to deteriorate if they experienced increases in blood pressure or heart rate that could be clinically important	X						

Pre existing cardiovascular disorders:§*							

Angina		X	X	X	X	X	

Life threatening arrhythmias and channelopathies		X	X	X	X	X	

(Moderate or) Severe hypertension		X	X	X	X	X	(X)

Heart failure		X	X	X	X	X	

Myocardial infarction		X	X	X	X	X	

Arterial occlusive disease		X	X	X	X	X	

Haemodynamically significant congenital heart disease		X	X	X	X	X	

Cardiomyopathies		X	X	X	X	X	

Known risk factors for cerebrovascular disorder		X					

Structural cardiac abnormality							X

Symptomatic cardiovascular disease							X

Advanced arteriosclerosis							X

Pre-existing (severe) cerebrovascular disorders	(X)**†**	X	X	X	X	X	

**Neurological**							

Tourette's syndrome/similar dystonias							X

*Categorised among pre-existing cardiovascular disorders and counted as one contraindication

§Cardiovascular conditions listed on the atomoxetine SPC as examples of severe cardiovascular disorders

†As specified and counted within cardiac contraindication for atomoxetine

ADHD: attention deficit hyperactivity disorder; MAOI: monoamine oxidase inhibitors; MPH: methylphenidate; SPC: Summary of Product Characteristics; UK: United Kingdom

Methylphenidate and atomoxetine are licensed in children aged 6 and over and dexamfetamine is licensed for children aged 3 and over with refractory HKD. None of the medications are licensed to treat adults although Strattera^® ^[12] and Concerta^® ^[13,14] may be used for transition into adulthood if initiated in childhood and a favourable response to treatment has been demonstrated.

Table 1 summarises when the products were originally licensed, the date of the latest authorisation and the date of the most recent substantive change to the SPC. The number of contraindications for each medication ranges from five (Strattera^®^) to 17 (Medikinet^® ^XL) (Table 2).

Following intervention by the EMA in January 2009 and the requirement for harmonisation of product information within the SPCs of products containing methylphenidate, the contraindications given within the SPCs for these medications are now almost the same. However, pronounced anacidity (absence of acidity, especially hydrochloric acid in the gastric juices) and use with H2 blockers or antacids is unique to Medikinet^® ^XL [15] while Ritalin^® ^[16] also contains an additional warning of "known risk factors for cerebrovascular disorders" which is not specified for the other short-acting methylphenidate formulation, Medikinet^® ^[17]. Although some similarities exist, the 14 contraindications for dexamfetamine [18] are quite different to those of atomoxetine and the varying forms of methylphenidate.

### UK national guidelines

The key national ADHD guidelines relating to the treatment of ADHD are from NICE (2008) [1] and SIGN (2009) [11]. The British National Formulary for Children (BNFC), which validates its information against emerging evidence, best-practice guidelines and a network of clinical experts, was also reviewed [19]. The British Association for the Psychopharmacology Transition Guidelines were considered beyond the scope of this paper as they focus on guiding clinicians who are specifically managing adolescents with ADHD in transition from children's services, and adults newly presenting with ADHD [20].

The NICE recommendations for treatment differ considerably from the information provided within the Indications for Treatment, Contraindications and Special Warnings and Precautions for Use sections in the SPCs. There is one significant conflict with the contraindications listed in all the methylphenidate SPCs; namely, NICE 72 does not state that suicidal tendencies are a contraindication to the use of methylphenidate. Eight important conflicts with the warnings and precautions in the SPCs of methylphenidate and atomoxetine were also identified (Table 3). These include advising different monitoring schedules of specific conditions, different potential side effects and physical well-being from those in the SPCs, advising potential use of methylphenidate in the presence of stimulant misuse or risk of diversion and recommendations for monitoring patients on atomoxetine for sexual dysfunction, which is not a requirement for children in the SPC. In addition, the use of methylphenidate is advised in adults even though at the time of the recommendation, no form of methylphenidate was approved for use in adults. However, on June 17^th ^2011, continuation use into adulthood was added to the SPC for Concerta^® ^[13,14]. Major areas of disparity arise around the recommendations made by NICE for monitoring of suicidal ideation and behaviours, self-harm and other psychiatric adverse events. Furthermore, recommendations in the methylphenidate SPC for monitoring for such psychiatric events are not included in NICE guidelines, while prominence is given to those for atomoxetine.

**Table 3:** Differences between NICE Guidelines and SPC Content

NICE Guideline Recommendation	Conflict (emphasis in bold reflects SPC)
Warn of suicidal problems and self harming behaviour with atomoxetine	Suicidal problems are a contraindication to the use of methylphenidate (omitted) Self-harm is not listed anywhere on the atomoxetine SPC Similar warnings are listed on SPCs for both atomoxetine and methylphenidate

Use MPH in adults	Most forms of methylphenidate are unlicensed. Atomoxetine and all strengths of Concerta are licensed in adults who were prescribed atomoxetine/Concerta in childhood and who continue to demonstrate a favourable response

Use methylphenidate or atomoxetine when stimulant misuse or risk of stimulant diversion are present	There are specific warnings on methylphenidate SPCs about risk of misuse, diversion and related issues. In high risk cases, the advice on methylphenidate SPCs is to use atomoxetine Atomoxetine has no warnings or precautions relating to drug misuse

In people with ADHD, heart rate and blood pressure should be monitored and recorded on a centile chart before and after each dose change and routinely every 3 months	Atomoxetine and methylphenidate-Blood pressure and pulse should be recorded on a centile chart at each adjustment of dose and then at least every 6 months Dexamfetamine-Blood pressure should be monitored at appropriate intervals in all patients taking dexamfetamine, especially those with hypertension

In people taking methylphenidate, atomoxetine, or dexamfetamine:	Growth and development should be monitored during treatment with atomoxetine
• height should be measured every 6 months in children and young people	Methylphenidate-Height, weight and appetite should be recorded at least 6 monthly with maintenance of a growth chart
• weight should be measured 3 and 6 months after drug treatment has started and every 6 months thereafter in children, young people and adults	Dexamfetamine-Height and weight should be carefully monitored in children as growth retardation may occur

In young people and adults, sexual dysfunction (i.e., erectile and ejaculatory dysfunction) and dysmenorrhoea should be monitored as potential side-effects of atomoxetine	This warning is not contained within the SPC for atomoxetine. Clinical trial data in adults shows sexual dysfunction as an undesirable effect. Post-marketing surveillance data in children, adolescents and adults list priapism and male genital pain as an undesirable effect

Use methylphenidate or atomoxetine with tics/Tourette's	In a controlled study of paediatric patients with ADHD and co morbid chronic motor tics or Tourette's Disorder, atomoxetine-treated patients did not experience worsening of tics compared to placebo-treated patients. There have been very rare post-marketing reports of tics in patients taking atomoxetine). Patients who are being treated for ADHD with atomoxetine should be monitored for the appearance or worsening of tics
	Methylphenidate is associated with the onset or exacerbation of motor and verbal tics. Worsening of Tourette's syndrome has also been reported. Family history should be assessed and clinical evaluation for tics or Tourette's syndrome in children should precede use of methylphenidate. Patients should be regularly monitored for the emergence or worsening of tics during treatment with methylphenidate. Monitoring should be at every adjustment of dose and then at least every 6 months or every visit

Use methylphenidate or atomoxetine with anxiety disorder	In two controlled studies (one in paediatric patients and one in adult patients) of patients with ADHD and co-morbid anxiety disorders, atomoxetine-treated patients did not experience worsening of anxiety compared to placebo-treated patients.
	There have been rare post-marketing reports of anxiety in patients taking atomoxetine. Patients who are being treated for ADHD with atomoxetine should be monitored for the appearance or worsening of anxiety symptoms.
	Methylphenidate is associated with the worsening of pre-existing anxiety, agitation or tension. Clinical evaluation for anxiety, agitation or tension should precede use of methylphenidate and patients should be regularly monitored for the emergence or worsening of these symptoms during treatment, at every adjustment of dose and then at least every 6 month or every visit

In particular, those treated with atomoxetine should be closely observed for agitation, irritability, suicidal thinking and self-harming behaviour, and unusual changes in behaviour, particularly during the initial months of treatment, or after a change in dose	Atomoxetine SPC-Treatment emergent agitation in children and adolescents without a prior history of psychotic illness or mania can be caused by atomoxetine at usual doses. Irritability is a common adverse event but has no monitoring requirement specifically
	Methylphenidate SPCs-Psychiatric disorders to monitor for... include (but are not limited to)... agitation, irritability. Methylphenidate is associated with the worsening of... agitation or tension. Clinical evaluation for... agitation or tension should precede use of methylphenidate and patients should be regularly monitored for the emergence or worsening of these symptoms during treatment, at every adjustment of dose and then at least every 6 month or every visit. Irritability is a common adverse effect of methylphenidate and patients receiving long-term therapy should be monitored for this amongst other psychiatric adverse effects.
	Suicidal ideation is an uncommon side effect on both methylphenidate and atomoxetine SPCs. Self harm is not mentioned on either SPC

ADHD: attention deficit hyperactivity disorder; MPH: methylphenidate; NICE: National Institute for Health and Clinical Excellence; SPC: Summary of Product Characteristics

The SIGN Guidelines contain several conflicts when compared with the Special Warnings and Precautions for Use in the SPCs of atomoxetine, methylphenidate and dexamfetamine. These relate to recommending use of methylphenidate when there are known risk factors, requirements for additional monitoring and warnings for atomoxetine not contained in the corresponding SPC and no mention of any monitoring requirements for dexamfetamine (Table 4).

**Table 4:** Differences between SIGN Guidelines and SPC Content

SIGN Guideline Recommendation	Conflict (emphasis in bold reflects SPC)
Use of modified release formulations or atomoxetine should be considered where there is likelihood of diversion	Particular warnings on methylphenidate SPC about risk of misuse, diversion and related issues. In high risk cases, advised on methylphenidate SPC to use atomoxetine. Atomoxetine has no such warnings or precautions

Where atomoxetine is prescribed, clinicians should review at least 6 monthly, including assessment of ongoing efficacy and adverse effects and measurement of growth, pulse and blood pressure (with correct cuff size) using appropriate centile charts	Atomoxetine-Where patients are continuing treatment with atomoxetine beyond 1 year, re-evaluation of the need for therapy by a specialist in the treatment of ADHD is recommended. Growth and development should be monitored during treatment with atomoxetine. Blood pressure and pulse should be recorded on a centile chart at each adjustment of dose and then at least every 6 months Methylphenidate- The physician who elects to use methylphenidate for extended periods (over 12 months) in children and adolescents with ADHD should periodically re-evaluate the long-term usefulness of the drug for the individual patient with trial periods off medication to assess the patient's functioning without pharmacotherapy. It is recommended that methylphenidate is de-challenged at least once yearly to assess the child's condition (preferable during times of school holidays). Improvement may be sustained when the drug is either temporarily or permanently discontinued. Blood pressure and pulse should be recorded on a centile chart at each adjustment of dose and then at least every 6 months. Height, weight and appetite should be recorded at least 6 monthly with maintenance of a growth chart
	Dexamfetamine- Blood pressure should be monitored at appropriate intervals in all patients taking dexamfetamine, especially those with hypertension. Height and weight should be carefully monitored in children as growth retardation may occur

(Atomoxetine)Additional monitoring is advised for those at risk of increased cardiovascular, hepatobiliary risk, seizure risk and potential suicidal ideation	Atomoxetine- Blood pressure and pulse should be recorded on a centile chart at each adjustment of dose and then at least every 6 months. No specified requirement for hepatic or seizure monitoring.
	Methylphenidate SPC-Patients on long-term therapy (i.e. over 12 months) must have careful ongoing monitoring according to the guidance in Sections 4.2 and 4.4 for cardiovascular status. **Cardiovascular status should be carefully monitored**

ADHD: attention deficit hyperactivity disorder; MPH: methylphenidate; SIGN: Scottish Intercollegiate Guidelines Network; SPC: Summary of Product Characteristics

The BNFC does not include the five contraindications for atomoxetine that are in the current SPC but reports side effects in children which have only been reported in clinical trials in adults; it also lists adverse events not included in the SPC. For example, sexual dysfunction is specifically listed as an adverse event associated with treatment with atomoxetine in a publication intended to be followed in the treatment of children but all reports of sexual dysfunction in clinical trials relate to the treatment of adults only and post-marketing surveillance reports of sexual dysfunction for adults, children and adolescents include only priapism and male genital pain. In addition, the BNFC does not report the full list of contraindications for dexamfetamine, omitting mention of Tourette's syndrome and other dystonias, sucrose and lactose intolerance. Rather it lists Tourette's syndrome as a caution not a contraindication. The 2010/2011 BNFC was not been updated to reflect the changes to the methylphenidate SPCs implemented following the CHMP review in 2009.

## Discussion

Since our original review of the methylphenidate SPC in 2008 [8], the CHMP has ensured a greater degree of consistency across the contraindications and other prescribing information listed in SPCs for the various formulations of methylphenidate. One formulation of methylphenidate, Equasym, has also been withdrawn and there have been three significant changes to the atomoxetine SPC. In terms of contraindications, there are numerically fewer in the atomoxetine SPC (five) than in any of the methylphenidate SPCs (maximum: 17), however, many of the specific contraindications in the methylphenidate SPCs can be found in the Special Warnings and Precautions for Use section of the atomoxetine SPC and recent changes to the contraindications for atomoxetine have made the prescribing information more similar. There remain some inconsistencies among the SPCs for methylphenidate although these are unlikely to be clinically significant. Given that atomoxetine and methylphenidate are different chemical compounds from different classes and have different pharmacokinetics and pharmacodynamics, it is unsurprising that the number and type of contraindications is also different. It is clearly important that clinicians are aware of the relative contraindications of all medications licensed to treat ADHD prior to prescribing for specific patients especially those with comorbidities which may be a contraindication for treatments.

There is disparity between the three sets of UK national guidance and the safety content and recommendations made in the SPCs for both atomoxetine and methylphenidate and to a lesser extent dexamfetamine. This lack of consistency and clarity within the information typically used to support prescribing decisions cannot help prescribers evaluate the suitability of specific medications for individual patients. It is of interest that the respective guidelines state that healthcare professionals are expected to take the SPC fully into account when exercising their clinical judgement and that the guidance does not override the individual responsibility of healthcare professionals to be informed by the SPC of any drugs [1].

There have been a number of initiatives in Europe and the United States over the last 10 years designed to increase the safety database in paediatric psychopharmacology. Specific requirements are detailed in the Food and Drug Administration Amendments Act of 2007 [21], while, in 2007, the EMA enacted a new "Paediatric Regulation" which mandates greater and more appropriate clinical trial activity and reporting of data during the development of medicines intended for use in children aged 0-17 years [22]. Of relevance to ADHD is the announcement from EMA's European Network of Paediatric Research Group in December 2010 of the 7^th ^European Commission's Framework Programme pharmacovigilance study into the long-term adverse effects of methylphenidate in ADHD (ADDUCE [Attention Deficit Hyperactivity Disorder Drugs Use Chronic Effects]) and a methodological study into the measurement of suicidality in paediatric clinical trials (STOP [Suicidality: Treatment Occurring in Paediatrics]) [23].

Suicidality is a key area of disparity between national guidance and SPCs. The SPC for atomoxetine specifically highlights the need for monitoring for the appearance or worsening of suicide-related behaviour and states that suicidal behaviours have occurred uncommonly in clinical trials. Suicide-related events are also listed as an uncommon Undesirable Effect on the SPC. The SPCs for methylphenidate list suicidal tendencies as a contraindication for usage, contain a warning relating to the emergence of suicidal behaviours and also list suicidal ideation as an uncommon Undesirable Effect. They also mandate monitoring for all psychiatric adverse events and this will include suicidal issues of all types. The dexamfetamine SPC states that any family history of suicide should be investigated prior to initiation of treatment as part of the screening for risk of bipolar disorder.

In contrast to the SPC content, the NICE guidelines warn clinicians of "...suicidal problems and self harming behaviour with atomoxetine" and make no mention of the risks associated with other treatments. The SIGN Guidelines repeat this disparity by stating the need to closely monitor patients on atomoxetine in particular for agitation, irritability, suicidal thinking and self-harming behaviour.

The atomoxetine suicidality data are derived from a retrospective analysis of atomoxetine usage in 14 paediatric trials which reported greater suicidal ideation with atomoxetine when compared with placebo (p = 0.016). In a cohort of 1,357 atomoxetine-treated subjects, there were five cases of suicidal ideation reported, no completed suicides and one suicidal attempt [24]. The same database also reported a meta-analysis of the suicide-related events reported from comparator studies of atomoxetine and methylphenidate and reported no difference in rates between these agents (Maentzel-Haentzel incidence difference -0.12 (95% confidence interval -0.62 to 0.38; p = 0.649). Hence, there would seem to be no specific data that suggest any differential suicidality between the treatments which is clearly not reflected in the National Guidelines. Clinical studies now include rating scales as well as pragmatic outcome measurements to assess items such as suicidality [25].

Similar inconsistencies arise with other sections of the SPCs. The NICE guidelines suggest using either atomoxetine or methylphenidate with co-morbid anxiety, but specifically recommend the need for observation for agitation or irritability only with atomoxetine despite a strong warning on the methylphenidate SPC relating to the need to clinically evaluate patients for agitation and irritability prior to the use of methylphenidate. The SPC also advises that patients should be regularly monitored for the emergence or worsening of these symptoms during treatment, at every adjustment of dose and then at least every 6 month or every visit. The atomoxetine SPC states that whilst treatment emergent agitation can be caused by atomoxetine at usual doses there are no specific monitoring recommendations around agitation and irritability. Methylphenidate is associated with the worsening of pre-existing anxiety, agitation or tension. Clinical evaluation for anxiety, agitation or tension should precede use of methylphenidate and patients should be regularly monitored for the emergence or worsening of these symptoms during treatment, at every adjustment of dose and then at least every 6 months or every visit. Recent changes to the atomoxetine SPC highlight that anxiety was not worsened in clinical trials but that patients being treated with atomoxetine should be monitored for the appearance or worsening of anxiety. The wording around the recommendations in the guidelines does not fully reflect the SPC content of either product.

A similar disparity exists for usage of either agent when tics or Tourette's syndrome are present. Both NICE and SIGN recommend use of either methylphenidate or atomoxetine although the SPCs have differences. The atomoxetine SPC reports that there was no worsening of tics or Tourette's in a placebo-controlled trial, very rare post-marketing reports of tics have been received and that patients should be monitored for the appearance or worsening of tics. The methylphenidate SPC states that methylphenidate is associated with the onset of tics, that both tics and Tourette's may worsen with methylphenidate treatment and that clinical evaluation and examination should precede methylphenidate treatment.

Other areas of debate include adverse events attributed to a drug. For example, in both National Guidelines, it is recommended that sexual dysfunction is monitored in atomoxetine patients (children and adults) but this requirement is not reflected in the SPC. The SPC contains no clinical trial data on sexual dysfunction specifically in children, only post-marketing surveillance data which includes children, adolescents and adults.

The disparity between these independent data sources may be complex for clinicians to interpret particularly when the NICE guidelines recommend use of methylphenidate in adults which has only been an approved indication for one form of methylphenidate (Concerta^®^) since June 2011 [13,14].

Stimulant misuse or diversion is another potential area of concern due to the nature of ADHD. No clear guidance is given in NICE despite clinical evidence that atomoxetine is not a drug of abuse. The methylphenidate SPC advises clinicians to prescribe atomoxetine in high risk cases and contains warnings and caveats about the potential for misuse or diversion. These distinctions in the SPCs are not translated into the guidelines.

It is frequently reported that co-morbidities are common in ADHD patients. A recent study on a cohort of 1,068 subjects with ADHD symptoms reported high rates of Generalised Anxiety Disorder (16.8%), dysthymic disorder (13.5%) and Major Depressive Disorder (3%) [26] when using the Diagnostic and Statistical Manual of Mental Disorders, volume 4 (DSM IV). Clinicians may thus be regularly evaluating drug choices in the presence of significant co-morbidities and any disparity between guidelines and SPC content may add to this complexity.

An important consideration is the balance of regulatory statements as contained in the SPC and the expertise of those routinely prescribing medications. Clinicians have had significant experience of using methylphenidate for many years in many children and as such have considerable expertise in weighing up potential tolerability and safety risks with the benefits of medication to individual patients. Relevant clinical publications, clinical expertise in using the medications and achieving positive results in cases that are contraindicated according to the SPC are highly influential in determining future drug use. There is experience of contraindicated medications being prescribed to patients with positive outcomes despite the potential risk of rare adverse events. The advantage of the regulatory statements is that they are based on cumulative case reports and as such reveal the rarer adverse events that would not otherwise be picked up in routine practice. As a consequence, appropriate levels of monitoring and precaution can be recommended for different products. These types of issue are evidenced by the level of debate in the scientific literature regarding the potential for methylphenidate to cause or worsen tics [1,10,20].

Clearly data are emerging rapidly in ADHD with the advent of new treatments and epidemiological research but national guidelines and SPCs cannot be updated as frequently as data emerge. For the period from January 2009 to April 2011, the search term "atomoxetine" in Pub Med generates 245 citations with at least 94 citations likely to contain clinical data, meta-analyses or reviews. The future safety of paediatric treatments are paramount and are likely to be advanced by the FDA and EMA regulations and the ability of large databases to address outcome measures of a more pragmatic kind. Some safety aspects may even be improved by drug treatment. Recent data on atomoxetine usage in a cohort of 13-16 year old children improving unhealthy dietary behaviours and physical activity as well as reducing behaviours contributing to unintentional injuries [27].

To be able to make prescribing decisions based on sound evidence, clinicians need to be aware of, and be familiar with, the different sources of information available to them. The SPC, as an independent document reflecting current knowledge, may be the best way of achieving this.

## Conclusion

Clinicians seeking guidance regarding the use of medications for ADHD in UK will find significant disparity between relevant national guidance and SPCs; these differences extend to licensed indications, contraindications, warnings and precautions and monitoring schedules. The contraindications sections within the SPC provide clear categorical statements for which the relevant medications should not be prescribed.

In view of the approval of the content of the SPC by the regulatory agency and the ongoing changes that take place to reflect current safety findings, many of which have been discussed in this manuscript, clinicians may be advised to consider the SPC as the ultimate guideline. The very recent decision by NICE not to update the current ADHD Guideline until 2014 may further increase the relevance of the respective SPCs.

## List of abbreviations

ADDUCE: ADHD Drugs Use Chronic Effects Programme; ADHD: Attention Deficit Hyperactivity Disorder; BNFC: British National Formulary for Children; CHMP: Committee for Medicinal Products for Human use; DSM-IV: Diagnostic and Statistical Manual of Mental Disorders Volume 4; EC: European Commission; EMA: European Medicines Agency; eMC: electronic Medicines Compendium; EU: European Union; FDA: Food and Drugs Administration; HKD: Hyperkinetic Disorder; MAH: Marketing Authorisation Holder; MHRA: Medicines and Healthcare products Regulatory Agency; NICE: National Institute for Health and Clinical Excellence; PSUR: Periodic Safety Update Report; SIGN: Scottish Intercollegiate Guidelines Network; SPC: Summary of Product Characteristics; STOP: Suicidality Treatment Occurring in Paediatrics; UK: United Kingdom.

## Competing interests

Nicola Savill and Chris Bushe are employees and shareholders of Eli Lilly and Company Ltd. Eli Lilly is the marketing authorisation holder and manufacturer of atomoxetine in the UK and has financed this manuscript.

## Authors' contributions

The paper has been written by NS and CB. The tables were compiled by NS and checked by CB. Both authors read and approved the final version of the manuscript.
